# Tuberculosis amidst COVID-19 in Pakistan: a massive threat of overlapping crises for the fragile healthcare systems

**DOI:** 10.1017/S0950268822000358

**Published:** 2022-02-22

**Authors:** Hashir Ali Awan, Abdul Moiz Sahito, Mahnoor Sukaina, Govinda Khatri, Summaiya Waheed, Fatima Sohail, Mohammad Mehedi Hasan

**Affiliations:** 1 Dow University of Health Sciences, Karachi, Pakistan; 2 Karachi Medical and Dental College, Karachi, Pakistan; 3 Department of Biochemistry and Molecular Biology, Faculty of Life Science, Mawlana Bhashani Science and Technology University, Tangail, Bangladesh; 4 Division of Infectious Diseases, The Red-Green Research Centre, BICCB, Dhaka, Bangladesh

**Keywords:** COVID-19, endemic disease, healthcare systems, Pakistan, tuberculosis

## Abstract

*Mycobacterium tuberculosis* is the cause of tuberculosis (TB), a granulomatous illness that mostly affects the lungs. Pakistan is one of the eight nations that accounts for two-thirds of all new cases of developing TB. TB has long been an endemic disease in Pakistan. According to the World Health Organization (WHO) estimates, the nation has over 500 000 incident TB infections per year, with a rising number of drug-resistant cases. Recently, the coexistence of COVID-19 and TB in Pakistan has provided doctors with a problem. Fever or chills, cough, shortness of breath or difficulty breathing are all signs of COVID-19. After SARS-CoV-2 infection, cough might persist for weeks or months and it is frequently accompanied by persistent tiredness, cognitive impairment, dyspnoea or pain – a group of long-term consequences known as post-COVID syndrome or protracted COVID. Coughing with mucus or blood, and coughing that continues over 2 months are indications of TB. The same clinical presentation features make it difficult for healthcare personnel to effectively evaluate the illness and prevent the spread of these fatal diseases. Pakistan lacks the necessary healthcare resources to tackle two contagious diseases at the same time. To counteract the sudden increase in TB cases, appropriate management and effective policies must be implemented. Thus, in order to prevent the spread of these infectious diseases, it is critical to recognise and address the problems that the healthcare sector faces, as well as to create an atmosphere in which the healthcare sector can function at its full potential.

## Introduction

*Mycobacterium tuberculosis* is responsible to cause tuberculosis (TB), a granulomatous disease that mainly affects the lungs and may disseminate to other organs such as the brain and cervical vertebra. Spinal TB (Pott's disease) is the most prevalent and one of the deadliest types of skeletal TB, accounting for 50% of all cases. Although the thoracolumbar junction appears to be the most commonly involved location in spinal TB, any portion of the spine might be impacted [1]. It spreads mainly through an airborne medium. Symptoms include fever, night sweats, chest pain, weight loss and severe cough with phlegm and may show up with haemoptysis. It has a high mortality rate after HIV infection, and therefore, poses a severe public health hazard [2, 3]. According to the World Health Organization (WHO), the highest number of TB incidents in 2019 was reported, including 44% of emerging new cases from South-East Asia [4]. Pakistan is one of the eight countries with two-third of current emerging TB cases. Globally, 30 countries with an ample burden of TB accounted for 87% of new cases in 2019. With 216 population, Pakistan is amongst some most populous countries with a 2.04% rise in statistics since 1995, it has shown a significant demand for health care systems to cater to a large population. The low-middle income status of Pakistan which is accompanied by a shortage of medical resources and lack of trained medical staff significantly creates a hindrance to access quality health benefits [5]. Amongst 195 countries globally, Pakistan ranks in the 154th position to access quality health facilities. The quality health infrastructure deficit is significantly explicit. Pakistan hosts a variety of communicable and non-communicable diseases that place its population at high risk of HIV, polio, dengue, malaria and TB [6, 7]. Recently, the state's healthcare system has had to deal with a rise in the incidence of fungal diseases, particularly cutaneous mucormycosis [8, 9]. Aside from that, dengue fever and measles have been shown to be on the rise [7]. A total of 4000 cases of mortality are observed each day globally due to TB infectious disease. The therapy to curb the disease is indeed an obstacle in low- and middle-income countries (LMICs). According to the studies, the south Asia inclusive of, Pakistan records a decline of 80% in diagnosis and testing, while a 75–80% decrement descried for the notification of new TB cases during the span of the COVID-19 pandemic. Furthermore, a 66.8% decline in routine Bacille Calmette-Guérin (BCG) vaccination enunciated during the catastrophic ongoing COVID-19 pandemic [10]. Moreover, the resources to eliminate TB consumed for COVID-19, including National TB Programme (NTP) workers, TB isolation wards, TB testing centres and physicians to direct the attention and resources for COVID-19 emergencies, hence majorly disrupting the management of TB at the national level [10]. The objective of this article is to highlight the rampant challenges faced during the TB management and catastrophic health crisis in one of the high TB burden countries, Pakistan, during the extant pandemic; and furthermore, suggestions and importance of TB health surveillance infrastructure of Pakistan to overcome the challenges.

## Burden and current status of TB in Pakistan during COVID-19 pandemic

**Fig. 1.**
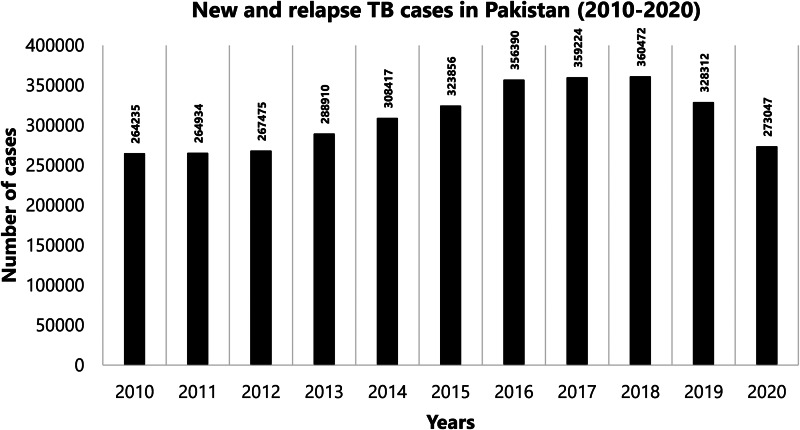
Trend of TB cases in Pakistan (2010–2020).

TB has long remained endemic in Pakistan. The WHO estimates over half a million incident TB cases every year in the country with an increasing proportion of drug-resistant cases [11]. The origin of nearly 61% TB cases in the Eastern Mediterranean WHO region is Pakistan, making it one of the leading contributors to the overall global TB burden [11]. According to WHO's Global Health Observatory, during the last decade, the number of new and relapse TB cases in Pakistan has remained relatively stable, with an average of over 312 222 cases per year (Fig. 1) [12]. However, a notable drop in 2020 was recorded [12, 13]. Latest provisional data from the WHO show 74 951 cases in the first quarter of 2021 [13].

Since late February 2020 till October 2021, Pakistan has battled multiple surges (or ‘waves’) of the COVID-19 pandemic and reported a cumulative total of nearly 1.25 million COVID-19 cases [14]. The coexistence of endemic diseases in LMICs like Pakistan with a global pandemic can prove to be a catastrophe of unprecedented proportions. The modes of transmission of TB and COVID-19 are distinct. While COVID-19 spreads predominantly through direct breathing and touching of objects on which droplets land when discharged by somebody with the illness during exhaling, sneezing, coughing and speaking, TB spreads through inhalation of TB bacilli that keep suspended in the air in droplet nuclei for many hours. Thus, in addition to handwashing, respiratory measures such as a face mask and social distance can have a discernible influence on global TB incidence during the present COVID-19 pandemic. Despite the COVID-19 safeguards, more people died from TB in 2020, with much fewer individuals detected, treated or given TB preventive medication than in 2019, and total spending on critical TB treatments dropping. The first issue is a loss of resources and a disruption in access to TB treatment. Many countries have diverted human, financial and other resources from TB control to the COVID-19 response, decreasing the availability of critical services. The second concern has been that some people have had difficulty obtaining medical assistance during lockdowns [15]. The acute burden and overwhelming nature of the COVID-19 crisis has invariably led to shifting of priorities and resources of already-underequipped and fragile healthcare infrastructure in many countries including Pakistan from other deadly diseases like TB [10, 16]. Pakistan's NTP also converted some laboratories dedicated to TB into additional COVID-19 testing centres [17]. In addition, a drop in TB cases during COVID-19 pandemic is not limited to Pakistan [6, 8]. Pakistan's neighbour, India, showed plummeting TB cases in the early days of the pandemic and a 21% decline from 2019 was reported worldwide in 2020 [10, 18]. Government-mandated lockdowns, disruption of routine out-patient services, rerouting of TB-specific facilities and fear of visiting hospitals all contributed to impaired case-detection of TB, letting multiple cases go undiagnosed [10, 18, 19]. Furthermore, due to COVID-19, stigma associated with respiratory diseases will potentially cause further isolation and refusal to report in many patients [20, 21]. In India, reviews have suggested widespread disruptions to surveillance, prevention and management of TB patients at all levels [22]. Importantly, studies with mathematical models focusing on countries with a high TB burden have predicted an 8–14% increase in TB-related deaths regardless of social distancing and a net increase in TB deaths is expected in all scenarios with any level of disruption in treatment provision [23].

The coincidence of COVID-19 and TB in Pakistan has presented a challenge to clinicians. Infection control and correct identification of the diseases are both of paramount importance. COVID-19 and TB are both predominantly pulmonary diseases [4]. The shared features in clinical presentation, such as cough, fever or shortness of breath, present a challenge to healthcare workers to adequately evaluate the condition and prevent the spread of both deadly diseases [20]. The mode of transmission of both diseases is extremely similar and explosive spread is possible in close contact and overcrowded settings [2, 5]. NTP in Pakistan has released extensive guidelines on outpatient evaluation of a patient presenting with symptoms that may overlap between COVID-19 and TB as it is highly likely for both patients to report to the same department [4, 17].

## Challenges and efforts

Although the WHO Directly Observed Treatment Short-course (DOTS) approach for TB was implemented and piloted in Pakistan from 1995 onwards, and the NTP achieved diverse success in managing TB cases for many years, in 2020 WHO asserted another public health emergency, COVID-19, which, alongside its potentially disastrous symptoms, impacted millions of individuals and became a burden on the public and health ministries.

Pakistan, as a low-middle-income nation with an underdeveloped healthcare system, a continual scarcity of medical personnel, rising medical expenditures, a lack of medical equipment and severe budgetary restrictions, is unable to combat the COVID-19 and TB co-epidemics [5]. Because of some overlapping symptoms between TB and COVID-19, misdiagnosis of TB occurs frequently, which contributes to the increased prognosis of TB cases; correspondingly, TB health services have decreased significantly due to a shortage of health care workers and hospitals as a result of the COVID-19 pandemic. Increasing patients' knowledge of clinical presentation, transmission and control methods of TB could lead to a significant decrease in disease prevalence, but a nationwide study found that the Pakistani population has insufficient knowledge of symptomatology, transmission, diagnosis and treatment of TB, as well as disease-related misapprehensions [24]. According to a qualitative study regarding the challenges faced by general practitioners (GP) in Pakistan, the significant proportion of GP were not aware of the appropriate sputum collection and how to consult a patient, the majority of GP were not aware of screening recommendations, they were not completely conscious of the definition of MDR-TB, the most of GP were thwarted when asked about management of TB in pregnant or lactating women, non-availability of tests were identified by some GPs, and none of the GP maintained a TB register for their patient [25]. As a result, the infrastructure of the healthcare system, as well as an absence of adequate expertise, poses a significant danger to Pakistan.

In Pakistan, the BCG vaccination is administered at birth to protect against TB. Some chemotherapy medicines used in Pakistan include isoniazid, rifampicin, pyrazinamide, ethambutol and streptomycin. A doctor's physical examination, laboratory investigation of the sputum, a chest X-ray, a blood test and an examination of fluid extracted from a lump are all used to diagnose TB [24]. Pakistan employs a Directly Observed Treatment method; according to WHO, it is a short course of therapy for TB and consists of identifying patients, treating them with medicines for 6–8 months and fostering adherence to a notoriously tough treatment regimen. Pakistan follows NTP in operation to diagnose, treat and prevent the spread of TB [17].

## Public health impact

Over the course of a year, persons with active TB can infect 5–15 other people through intimate contact. Without adequate treatment, 45% of HIV-negative TB patients and virtually all HIV-positive TB patients would die [4]. As drug resistance grows, the options available to tackle it become fewer and fewer. Unfortunately, complacency in the face of TB is also the reason that few novel therapies are being discovered. Unlike therapy for drug-sensitive TB, which is nearly always 100% successful when done correctly, treating drug-resistant TB is significantly more challenging. Treatment for severely drug-resistant TB can take up to 2 years and includes a cocktail of progressively unpleasant medicines with side effects ranging from nausea to irreversible hearing loss. When it comes to health-related quality of life in persons with TB, it was discovered that it is much poorer than in those without TB [26].

## Action plans

The strategies to tackle this threat involve:

1. Allowing a convenient and close provision of healthcare services to the people

This can be achieved by delivering medicines at their doorsteps and for longer duration and free of cost, or building community medicine collection points for providing medicines [27, 28]. This would reduce the number of visits and risk of exposure. Younger attendants (below 50 years of age) could on behalf of their patients collect the medicines and shifts could be arranged with four visitors at the maximum to minimise the exposure. Telemedicine services that encourage compliance to medication, act as a means to tackle any emergencies such as an adverse reaction and are a source of counselling should be introduced [29]. Establishing a link between private healthcare professionals (HCP) and the Pakistan NTP, strengthening treatment and reporting of TB patients, and improving TB screening, including simultaneous screening for TB and COVID-19 in patients with cough and fever, could help us deal with two problems at the same time. A door-to-door collection of sputum can improve TB screening.

1. Empowering the primary care setup

By training the HCPs regarding infection and prevention control measures and correct use of personal protective equipment [28]. The diagnostic and treatment centres for TB should be built and maintained according to airborne infection control standards that include large open fronts, exhausts for ventilation and air circulation, ultraviolet germicidal irradiation lights and designated sputum expectoration areas [27]. To reduce the risk of COVID spread, staff should be rotated in shifts, working hours should be reduced, N-95 masks for staff and surgical masks for visitors should be provided, physical separation in waiting areas should be ensured, easy access to disinfectants should be provided and regular hand washing practises should be implemented. The use of electronic medical records to track diagnostic tests and therapy should be encouraged.

1. Managing the stigma and fears

TB patients may be prone to stigma due to the similar nature of symptoms with COVID-19. TB is termed as a ‘great imitator’ due to its initial presentation with fever and respiratory symptoms [30] and many patients have been treated for COVID-19 despite a negative rt-PCR result until a sputum test confirmed TB. Thus, it is important to rightly introduce TB interventions in the communities by using the help and expertise of lady health workers (LHWS) especially at rural level [28]. To avoid the spread of misconceptions about COVID-19 and TB, people should be taught about both through awareness campaigns and at congregational locations, and this community-level education encourages a person-centred approach to TB prevention.

As per precedent of SARS-CoV-2 and *M. tuberculosis* coexisting, there is a reasonable threat that TB and COVID-19 may coexist [31]. Evidence from various countries including Italy has been reported to show varying clinical presentations of a COVID-19 and TB infection co-occurring in individuals [32–34]. Also, due to the threat of TB or COVID-19 being misdiagnosed as the other, it is critical to screen and test for both disorders [30]. As WHO recommends for multi-disease testing, an integrated method might be utilised to identify and isolate both infections. Finally, in order to prevent paediatric TB, it is critical to guarantee the unhindered delivery of the only TB vaccination, the BCG vaccine.

## Conclusion

The COVID-19 pandemic has presented a great challenge globally. Developing countries such as Pakistan have always been in a struggle to fight infectious diseases such as polio and TB. Amidst the ongoing battle against preexisting infectious diseases, this new infection could reduce the attention necessary for them and present with certain challenges like reduction in TB services due to lockdown and social distancing measures, which would automatically affect the screening, diagnosis and treatment of TB. Thus, in order to control the spread of these infectious diseases, it is crucial to identify and address these challenges by first allowing an easy access to healthcare facilities such as delivering TB medicines for long periods at door steps of patients or setting up collection points for them, by creating telemedicine services; second by strengthening the healthcare system to rightfully fight the infections simultaneously; and last, by addressing the stigma through awareness campaigns and educating people. Although it may seem daunting, a correct and collective approach with a correct mindset along with the aforementioned actions if taken can combat with the two diseases simultaneously and thus, prevent a major havoc to the healthcare system of Pakistan.

## Data Availability

Data sharing is not applicable to this article as no new data were created or analysed in this study.

## References

[1] Rasouli MR (2012) Spinal tuberculosis: diagnosis and management. Asian Spine Journal 6, 294. 23275816 10.4184/asj.2012.6.4.294 PMC3530707

[2] Fogel N (2015) Tuberculosis: a disease without boundaries. Tuberculosis 95, 527–531. 26198113 10.1016/j.tube.2015.05.017

[3] Islam Z (2021) Tuberculosis behind bars in Latin America and Caribbean: a growing public health crisis. Infection Control & Hospital Epidemiology, 1–2. doi: 10.1017/ICE.2021.424 34612189

[4] World Health Organization (2021). Tuberculosis. https://www.who.int/news-room/fact-sheets/detail/tuberculosis (Accessed 11 October 2021).

[5] Awan UA (2021) COVID-19 and tuberculosis overlapping epidemics: a holistic review from Pakistan. Journal of Medical Virology 93, 2573–2575. 33295641 10.1002/jmv.26714

[6] Hussain R and Arif S (2021) Universal health coverage and COVID-19: recent developments and implications. Journal of Pharmaceutical Policy and Practice 14, 1–4. 33568229 10.1186/s40545-021-00306-x PMC7874562

[7] Yousaf A (2021) Dengue, measles, and COVID-19: a threefold challenge to public health security in Pakistan. Ethics, Medicine and Public Health 19, 100704. 10.1016/j.jemep.2021.100704 PMC8249682 34230890

[8] Asri S (2021) The risk of cutaneous mucormycosis associated with COVID-19: a perspective from Pakistan. International Journal of Health Planning and Management. doi: 10.1002/HPM.3311 PMC8653268 34476830

[9] Ghazi BK (2021) Rampant increase in cases of mucormycosis in India and Pakistan: a serious cause for concern during the ongoing COVID-19 pandemic. American Journal of Tropical Medicine and Hygiene 1. doi: 10.4269/AJTMH.21-0608 PMC8592182 34460426

[10] Malik AA (2020) Tuberculosis control and care in the era of COVID-19. Health Policy and Planning 35, 1130–1132. 32832996 10.1093/heapol/czaa109 PMC7499582

[11] World Health Organization (2021) Regional Office for the Eastern Mediterranean (EMRO). Pakistan, programme areas, tuberculosis. http://www.emro.who.int/pak/programmes/stop-tuberculosis.html (Accessed 11 October 2021).

[12] World Health Organization (2021) The Global Health Observatory (GHO). TB: new and relapse cases; Pakistan. https://www.who.int/data/gho/data/indicators/indicator-details/GHO/tuberculosis---new-and-relapse-cases (Accessed 11 October 2021).

[13] World Health Organization (2021) The Global Health Observatory (GHO). Provisional number of new and relapse TB cases per quarter, 2020 & 2021. https://worldhealthorg.shinyapps.io/tb_pronto/ (Accessed 11 October 2021).

[14] Dong E, Du H and Gardner L (2020) An interactive web-based dashboard to track COVID-19 in real time. Lancet Infectious Diseases 20, 533–534. 32087114 10.1016/S1473-3099(20)30120-1 PMC7159018

[15] World Health Organization (2021) Tuberculosis deaths rise for the first time in more than a decade due to the COVID-19 pandemic. https://www.who.int/news/item/14-10-2021-tuberculosis-deaths-rise-for-the-first-time-in-more-than-a-decade-due-to-the-covid-19-pandemic (Accessed 4 February 2022).

[16] Roberts L (2021) How COVID hurt the fight against other dangerous diseases. Nature 592, 502–504. 33883727 10.1038/d41586-021-01022-x

[17] National TB Control Program, Pakistan. Resource Center (2021). https://ntp.gov.pk/resource-center/ (Accessed 11 October 2021).

[18] Bardhan M (2021) Tuberculosis amidst COVID-19 pandemic in India: unspoken challenges and the way forward. Tropical Medicine and Health 49, 84. 34674772 10.1186/s41182-021-00377-1 PMC8528656

[19] Khan FMA (2021) Resurgence of tuberculosis amid COVID-19 in Peru: associated risk factors and recommendations. International Journal of Health Planning and Management. doi: 10.1002/HPM.3291 34318523

[20] Fatima R and Yaqoob A (2020) In reply: how TB and COVID-19 compare: an opportunity to integrate both control programmes. The International Journal of Tuberculosis and Lung Disease 24, 1227–1228. 33172538 10.5588/ijtld.20.0571

[21] Islam M (2020) Extensively drug-resistant tuberculosis in the time of COVID-19 – how has the landscape changed for Pakistan? Disaster Medicine and Public Health Preparedness 14, 1. 10.1017/dmp.2020.230 PMC7371846 32635960

[22] Jain VK (2020) Tuberculosis in the era of COVID-19 in India. Diabetology & Metabolic Syndrome 14, 1439–1443. 10.1016/j.dsx.2020.07.034 PMC7387287 32755848

[23] Finn McQuaid C (2020) The potential impact of COVID-19-related disruption on tuberculosis burden. European Respiratory Journal 56. doi: 10.1183/13993003.01718-2020 PMC7278504 32513784

[24] Sara Ijaz Gilani MK (2012) Perception of tuberculosis in Pakistan: findings of a nation-wide survey. Journal of Pakistan Medical Association. https://pubmed.ncbi.nlm.nih.gov/22755370/ (Accessed 11 October 2021). 22755370

[25] Saima Perwaiz Iqbal MR (2013) Challenges faced by general practitioners in Pakistan in management of tuberculosis: a qualitative study. Rawal Medical Journal 38, 249–252.

[26] Wong YJ (2021) Impact of latent tuberculosis infection on health and wellbeing: a systematic review and meta-analysis. European Respiratory Review 30, 1–11. 10.1183/16000617.0260-2020 PMC9489106 33408089

[27] Jamal WZ (2020) COVID-19: ensuring continuity of TB services in the private sector. The International Journal of Tuberculosis and Lung Disease 24, 870–872. 32912398 10.5588/ijtld.20.0400

[28] Fatima R (2021) Building better tuberculosis control systems in a post-COVID world: learning from Pakistan during the COVID-19 pandemic. International Journal of Infectious Diseases. doi: 10.1016/J.IJID.2021.03.026 PMC7968149 33744479

[29] Farooqi M (2021) The revival of telemedicine in the age of COVID-19: benefits and impediments for Pakistan. Annals of Medicine and Surgery 69, 102740. 34457264 10.1016/j.amsu.2021.102740 PMC8379814

[30] Sarinoglu RC (2020) Tuberculosis and COVID-19: an overlapping situation during pandemic. The Journal of Infection in Developing Countries 14, 721–725. 32794460 10.3855/jidc.13152

[31] Bandyopadhyay A (2020) COVID-19 and tuberculosis co-infection: a neglected paradigm. Monaldi Archives for Chest Disease 90, 518–522. 10.4081/monaldi.2020.1437 32885625

[32] Tadolini M (2020) Active tuberculosis, sequelae and COVID-19 co-infection: first cohort of 49 cases. European Respiratory Journal 56. doi: 10.1183/13993003.01398-2020 PMC7251245 32457198

[33] Stochino C (2020) Clinical characteristics of COVID-19 and active tuberculosis co-infection in an Italian reference hospital. European Respiratory Journal 56. doi: 10.1183/13993003.01708-2020 PMC7263070 32482787

[34] Kozinska M (2021) TB and COVID-19 coinfection. The International Journal of Tuberculosis and Lung Disease 25, 776. 34802504 10.5588/ijtld.21.0358

